# Vitamin D deficiency exacerbates COPD-like characteristics in the lungs of cigarette smoke-exposed mice

**DOI:** 10.1186/s12931-015-0271-x

**Published:** 2015-09-16

**Authors:** Nele Heulens, Hannelie Korf, Nele Cielen, Elien De Smidt, Karen Maes, Conny Gysemans, Erik Verbeken, Ghislaine Gayan-Ramirez, Chantal Mathieu, Wim Janssens

**Affiliations:** Laboratory of Respiratory Diseases, Department of Clinical and Experimental Medicine, Katholieke Universiteit Leuven, Herestraat 49, 3000 Leuven, Belgium; Laboratory of Clinical and Experimental Endocrinology, Department of Clinical and Experimental Medicine, Katholieke Universiteit Leuven, Herestraat 49, 3000 Leuven, Belgium; Translational Cell and Tissue Research, Department of Imaging and Pathology, Katholieke Universiteit Leuven, Minderbroederstraat 12, 3000 Leuven, Belgium

## Abstract

**Background:**

Chronic obstructive pulmonary disease (COPD) is characterized by excessive inflammation and disturbed bacterial clearance in the airways. Although cigarette smoke (CS) exposure poses a major risk, vitamin D deficiency could potentially contribute to COPD progression. Many *in vitro* studies demonstrate important anti-inflammatory and antibacterial effects of vitamin D, but a direct contribution of vitamin D deficiency to COPD onset and disease progression has not been explored.

**Methods:**

In the current study, we used a murine experimental model to investigate the combined effect of vitamin D deficiency and CS exposure on the development of COPD-like characteristics. Therefore, vitamin D deficient or control mice were exposed to CS or ambient air for a period of 6 (subacute) or 12 weeks (chronic). Besides lung function and structure measurements, we performed an in depth analysis of the size and composition of the cellular infiltrate in the airways and lung parenchyma and tested the *ex vivo* phagocytic and oxidative burst capacity of alveolar macrophages.

**Results:**

Vitamin D deficient mice exhibited an accelerated lung function decline following CS exposure compared to control mice. Furthermore, early signs of emphysema were only observed in CS-exposed vitamin D deficient mice, which was accompanied by elevated levels of MMP-12 in the lung. Vitamin D deficient mice showed exacerbated infiltration of inflammatory cells in the airways and lung parenchyma after both subacute and chronic CS exposure compared to control mice. Furthermore, elevated levels of typical proinflammatory cytokines and chemokines could be detected in the bronchoalveolar lavage fluid (KC and TNF-α) and lung tissue (IP-10, MCP-1, IL-12) of CS-exposed vitamin D deficient mice compared to control mice. Finally, although CS greatly impaired the *ex vivo* phagocytic and oxidative burst function of alveolar macrophages, vitamin D deficient mice did not feature an additional defect.

**Conclusions:**

Our data demonstrate that vitamin D deficiency both accelerates and aggravates the development of characteristic disease features of COPD. As vitamin D deficiency is highly prevalent, large randomized trials exploring effects of vitamin D supplementation on lung function decline and COPD onset are needed.

**Electronic supplementary material:**

The online version of this article (doi:10.1186/s12931-015-0271-x) contains supplementary material, which is available to authorized users.

## Background

Chronic obstructive pulmonary disease (COPD) is a chronic disease characterized by a progressive expiratory airflow limitation and is associated with chronic inflammation in the airways and lung parenchyma [1]. In the majority of cases, this inflammatory response in COPD is initiated by long-term exposure to cigarette smoke (CS), which triggers a series of events that damage the airways and terminal airspaces, leading to lung function decline and emphysema. With progression of the disease, COPD patients become more susceptible to exacerbations, a flaring-up of the disease primarily induced by respiratory bacterial and viral infections [2]. Exacerbations are an important cause of hospitalization, reduced quality of life and mortality.

Interestingly, epidemiological evidence suggests a role for vitamin D deficiency in COPD onset and progression. We and others previously demonstrated that vitamin D deficiency (defined as serum 25-hydroxyvitamin D (25OHD) < 20 ng/ml) is highly prevalent in COPD patients and furthermore correlates with disease severity (as assessed by the forced expiratory volume in 1 s (FEV1)) [3–5]. In addition, associations have been made between low serum 25OHD levels and characteristic disease features of COPD, including reduced lung function (FEV1 and forced vital capacity (FVC)) [3, 6, 7], faster lung function decline [8], severity of CT-defined emphysema [9] and risk at COPD exacerbations [10]. However, evidence from epidemiological studies remains conflicting as different negative studies have been published showing absence of associations [11–14]. The controversy remains high as many of the pathogenic processes of COPD progression, such as pulmonary inflammation, oxidative stress, parenchymal destruction (emphysema) as well as defective antibacterial responses can be counteracted *in vitro* by the active form of vitamin D (1,25-dihydroxyvitamin D (1,25(OH)_2_D)) [15]. For example, 1,25(OH)_2_D may promote antibacterial defense by enhancing phagocytosis, oxidative burst, chemotaxis as well as production of antimicrobial peptides [16, 17], while impeding excessive production of inflammatory cytokines in airway epithelial cells, monocytes, macrophages and dendritic cells [18–22]. 1,25(OH)_2_D also inhibits the expression of several matrix metalloproteinases (MMPs) [23, 24], which contribute to parenchymal destruction.

Taken together, the direct impact of vitamin D deficiency on CS-induced inflammation and the development of COPD remains unclear. To directly address these issues, we investigated the effect of vitamin D deficiency on i) lung function and structure, ii) pulmonary inflammation and iii) alveolar macrophage antibacterial function in a mouse model of subacute and chronic CS exposure.

## Methods

### Animals

C57Bl/6J mice were purchased from Harlan. 3-weeks-old male and female C57Bl/6J mice were fed with either a vitamin D deficient diet (containing <100 IU/kg) or a control diet (1000 IU/kg) (Ssniff) (Bio-services; Netherlands) and kept in UV light-free surroundings. By the age of 8 weeks, breeding pairs were formed. Their male offspring was used in this study and received the same vitamin D deficient or control diet. All mice were housed in UV light-free surroundings in a conventional animal house with a 12/12 h light–dark cycle. Animals were placed in filter-top cages and supplied with pelleted food and water ad libitum. All experiments were approved by the Ethical Committee of Animal Experiments of the KU Leuven (P099/2011).

### Cigarette smoke exposure

Vitamin D deficient or control mice were randomly divided into 2 separate groups: CS-exposed groups and air-exposed groups. Animals were exposed to CS (3R4F research cigarettes with filter, Kentucky Tobacco Research and Development Center, University of Kentucky) using a nose-only exposure system (InExpose System, Scireq). Mice were placed in soft restraints and connected to a smoke-exposure tower. Computer-controlled puffs were generated every minute, leading to 10 s of CS exposure followed by 50 s of fresh air. At 8 weeks of age, mice were acclimatized to CS by gradually increasing the amount of cigarettes during the first 2 weeks of the experiment. Afterwards, animals were exposed to 4 cigarettes, twice a day, 5 days per week, for a period of 6 weeks (subacute) or 12 weeks (chronic). Similarly, control mice were put into soft restraints, but were exposed to ambient air for the same period of time. The total particulate density was measured daily and mice were weighed weekly to monitor health conditions of the animals.

### Lung function

Twenty-four hours after the last CS exposure, mice were anaesthetized intraperitoneally with a mixture of xylazine (8,5 mg/kg, Rompun, Bayer, Belgium) and ketamine (130 mg/kg, Ketalar, Pfizer, Belgium). Tracheotomy was performed with a standard catheter (CNS5002) provided by the Buxco system. Mice were placed in a whole-body plethysmograph and connected to a computer-controlled ventilator (Buxco-Forced Pulmonary Maneuvers). Three different maneuvers were performed: the Boyle’s law FRC maneuver, the quasi-static pressure volume maneuver and the fast flow volume maneuver, as previously described [25]. The Boyle’s law FRC maneuver measures the functional residual capacity (FRC), while the quasi-static pressure volume maneuver measures static lung volumes such as total lung capacity (TLC), inspiratory capacity (IC), vital capacity (VC) as well as the quasi-static lung compliance (Cchord), which is defined as the volume-pressure ratio at 50 % of expiration. The fast volume maneuver determines dynamic lung volumes, such as FVC and forced expiratory volume in 100 ms (FEV0.1).

### Bronchoalveolar lavage and lung histology

After lung function measurements, mice were euthanized by an intracardiac administration of pentobarbital (Ceva, Brussels, Belgium). Bronchoalveolar lavage (BAL) was performed with Hanks Balanced Salt Solution supplemented with 10 mM HEPES. The supernatant of the first BAL fraction was stored at −80 °C for cytokine and chemokine analysis. Total cell count was performed using a Neubauer hemocytometer. After ligation of the right lung, the heart-left lung block was excised and fixed in 6 % paraformaldehyde at a constant hydrostatic pressure of 25 cm fluid column for 24 h. After dehydration and embedding in paraffin, sagittal sections were stained with H&E. Airspace enlargement (emphysema) was quantified by measuring the mean linear intercept (Lm) in 15 randomly selected fields per lung slide, at 200X magnification. The Lm was calculated as the total length of the grid lines x random fields divided by the sum of the alveolar intercepts. A pathologist (E.V) semi-quantitatively scored the parenchymal inflammation as well as the severity of irregular airspace enlargement on histological lung slices in a blinded manner.

### Flowcytometric analysis of BAL cells

BAL cells were stained with fluorochrome-labeled monoclonal antibodies against CD4, CD8, CD11b, CD11c, Ly6C and Ly6G (eBioscience) for 20 min at 4 °C. After washing, samples were acquired on a Gallios flow cytometer (Beckman Coulter, Brea, CA, USA) and analyzed with the Kaluza software (Beckman Coulter). Alveolar macrophages were identified as the CD11c^+^autofluorescent^high^ cell population, whereas neutrophils were identified as the CD11c^−^CD11b^+^Ly6G^+^ cell population.

### Alveolar macrophage antibacterial function

The *ex vivo* phagocytic and oxidative burst capacity of alveolar macrophages was assessed by flow cytometry using an adaptation of the commercial kit BURSTTEST (Orpegen Pharma, Heidelberg, Germany). Briefly, BAL cells were incubated for 2 h at 37 °C with *pH*-rodoRed-labeled *E. coli* bacteria (*pH*rodo™ Red *E. coli* Bioparticles Phagocytosis kit for flow cytometry, Invitrogen, Lennik, Belgium), previously opsonized with *E. coli* BioParticles opsonizing reagent (Invitrogen). After a 2-h incubation at 37 °C, the fluorogenic substrate rhodamine was added. Following 20 min of incubation at 37 °C, surface staining of the alveolar macrophages was performed as described above. Samples were acquired on a Gallios flow cytometer (Beckman Coulter) and analyzed with the Kaluza software (Beckman Coulter).

### Pro-inflammatory cytokines and chemokines in the BAL fluid

Levels of IL-12p70, IL-6, KC, IL-10 and TNF-α in the BAL fluid were measured using the mouse proinflammatory panel 1 kit (Mesoscale Discovery, Gaithersburg, MD, USA), according to the manufacturer’s instructions. Data were acquired on a MESO QuickPlex SQ 120 system and analyzed with the MSD Discovery Workbench software (Mesoscale Discovery).

### Quantification of relative mRNA levels

The right lung was snap-frozen in liquid nitrogen and stored at −80 °C. Total RNA was extracted using the RNeasy mini kit (Qiagen, Leudsen, Netherlands). A constant amount of 1 μg of RNA was reverse transcribed with Superscript III reverse transcriptase (Invitrogen) and 5 mM oligo(dT)_16_ at 42 °C for 80 min. The qPCR amplification reaction was performed on a StepOne™ real-time PCR system (Applied Biosystems, Carlsbad, CA, USA) using the Fast (SYBR Green) Master Mix or the TaqMan Fast Universal PCR Master Mix (Applied Biosystems). The primer and Taqman probe sequences used are shown in Table 1. Ribosomal protein L27 (RPL27) was used as housekeeping gene. Data were analyzed using the comparative cycle threshold (Ct) method.

**Table 1:** Primers used for quantitative PCR analysis

Target	Sequence
RPL27	5′-GTCGAGATGGGCAAGTTCAT-3′ (FW)
	5′-TTCTTCACGATGACGGCTTT-3′ (RV)
IP-10	5′-GCCGTCATTTTCTGCCTCAT-3′ (FW)
	5′-GCTTCCCTATGGCCCTCATT-3′ (RV)
	5′-TCTCGCAAGGACGGTCCGCTG-3′ (TP)
MCP-1	5′-CTTCTGGGCCTGCTGTTCA-3′ (FW)
	5′-CCAGCCTACTCATTGGGATCA -3′ (RV)
	5′-CTCAGCCAGATGCAGTTAACGCCCC-3′ (TP)
IL12p40	5′-GGAAGCACGGCAGCAGAATA-3′ (FW)
	5′-AACTTGAGGGAGAAGTAGGAATGG-3′ (RV)
	5′-CATCATCAAACCAGACCCGCCCAA-3′ (TP)
MMP-8	5′-CTTTCAACCAGGCCAAGGTA-3′ (FW)
	5′-GAGCAGCCACGAGAAATAGG-3′ (RV)
MMP-9	5′-TTCCCCAAAGACCTGAAAAC-3′ (FW)
	5′-TGCTTCTCTCCCATCATCTG-3′ (RV)
MMP-12	5′-TTTTGATGGCAAAGGTGGTA-3′ (FW)
	5′-GCCTCATCAAAATGTGCATC-3′ (RV)
TIMP-1	5′-GTGGGAAATGCCGCAGAT-3′ (FW)
	5′-GGGCATATCCACAGAGGCTTT-3′ (RV)

*FW* forward primer, *RV* reverse primer, *TP* Taqman probe

### Serum measurements

Blood was collected from the vena cava. Serum 25OHD levels were measured by liquid-phase radioimmunoassay (Diasorin, Stillwater, MN, USA). Calcium levels were determined using an adaption of the Calcium Gen.2 kit (Roche Diagnostics, Vilvoorde, Belgium). The intra-assay coefficient of variation for 25OHD and calcium measurements were 2.38 and 2.23 % respectively. The lower detection limits were 1.5 ng/ml for 25OHD measurements and 0.8 mg/dl for calcium measurements.

### Statistical analysis

Data were analyzed using SAS software version 9.3 and are presented as mean ± SEM. Statistical analysis was performed using a two-way ANOVA at each timepoint with a Tukey-Kramer *post hoc* test for multiple group comparison. Differences were considered significant when p-values were less than 0.05.

## Results

### The effect of vitamin D deficiency on serum 25OHD and calcium levels

Lifelong feeding of C57Bl/6J mice with a control diet (vitamin D sufficient) resulted in serum 25OHD concentrations of approximately 75–90 ng/ml, a level that is similar to all our mouse colonies housed and purchased by our facility (Table 2). Conversely, feeding of mice with a vitamin D deficient diet from *in utero* until adulthood significantly lowered serum 25OHD concentrations to 17–20 ng/ml. Serum calcium levels did not differ between vitamin D deficient or control mice (Table 2). Moreover, both serum 25OHD and calcium levels were similar after 6 and 12 weeks. None of the above parameters were affected by CS exposure (Table 2).

**Table 2:** Serum 25-hydroxyvitamin D and calcium levels

	Vitamin D deficient		Control	
	*Air-exposed*	*CS-exposed*	*Air-exposed*	*CS-exposed*
25OHD (ng/ml)	19.76 ± 1.87^a^	17.37 ± 2.68^b^	76.30 ± 3.15	86.76 ± 4.03
Calcium (mg/dl)	9.71 ± 0.62	10.97 ± 0.47	10.24 ± 0.26	9.09 ± 1.21

At the age of 8 weeks, C57Bl/6J vitamin D deficient and control mice were exposed to CS or ambient air for a period of 6 or 12 weeks. Data show results after 12 weeks and are represented as mean ± SEM.^a^ *p* < 0.0001 vs air-exposed control;^b^ *p* < 0.0001 vs CS-exposed control

### Vitamin D deficiency accelerates lung function decline following CS exposure

**Fig. 1:**
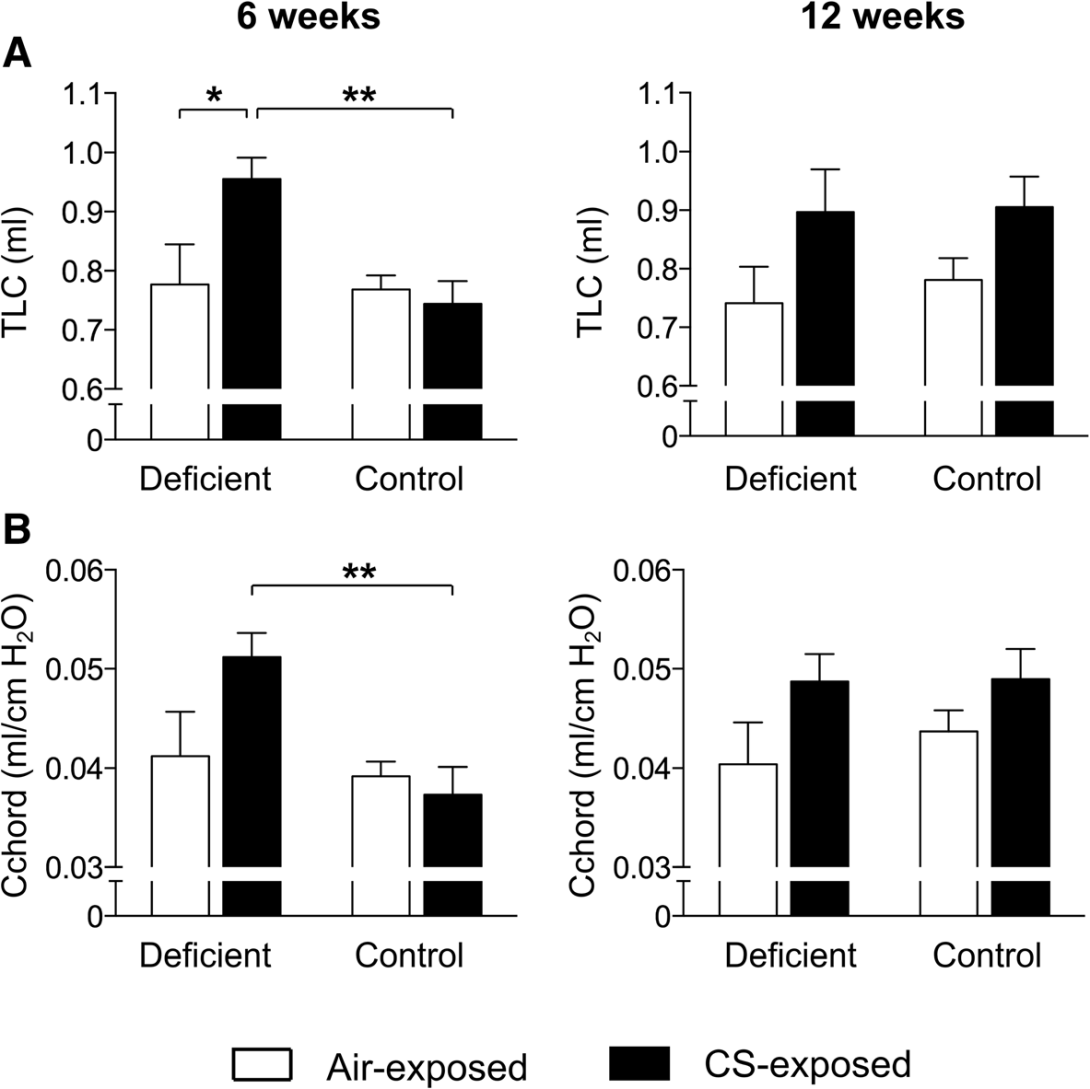
Effect of vitamin D deficiency on lung function parameters in air- and CS-exposed mice after 6 and 12 weeks. At the age of 8 weeks, C57Bl/6J vitamin D deficient and control mice were exposed to CS or ambient air for a period of 6 or 12 weeks. Lung function was measured with whole-body plethysmography after 6 and 12 weeks of smoking. **a** The total lung capacity (TLC) and **b** lung compliance (Cchord). *n* = 10–12 per group per timepoint; mean ± SEM; *p < 0.05, **p < 0.01

To investigate the effect of vitamin D deficiency on CS-induced aberrations in the lungs, vitamin D deficient or control mice entered a daily CS- or ambient air exposure regimen for 6 weeks (subacute) or 12 weeks (chronic) starting at the age of 8 weeks. After 6 weeks of CS exposure, parameters indicating lung hyperinflation (TLC, compliance, IC, VC, FVC and FEV0.1) remained unchanged in control mice (Fig. 1 + Additional file 1). However, in vitamin D deficient mice, signs of lung hyperinflation were already detected after 6 weeks of CS exposure. This was evident from a 23 % increase in TLC and a 24 % increase in compliance (Fig. 1a-b). Similar trends were observed for IC, VC, FEV0.1 and FVC (Additional file 1). Only after chronic CS exposure, a trend towards hyperinflation of the lungs was observed in control mice, as shown by an increase in TLC (16 %) and compliance (12 %) (Fig. 1) as well as IC, VC, FVC and FEV0.1 (Additional file 1). No additional differences were observed after 12 weeks of smoking in vitamin D deficient mice.

### Vitamin D deficient mice feature early signs of emphysema accompanied by elevated MMP-12 expression levels following CS exposure

**Fig. 2:**
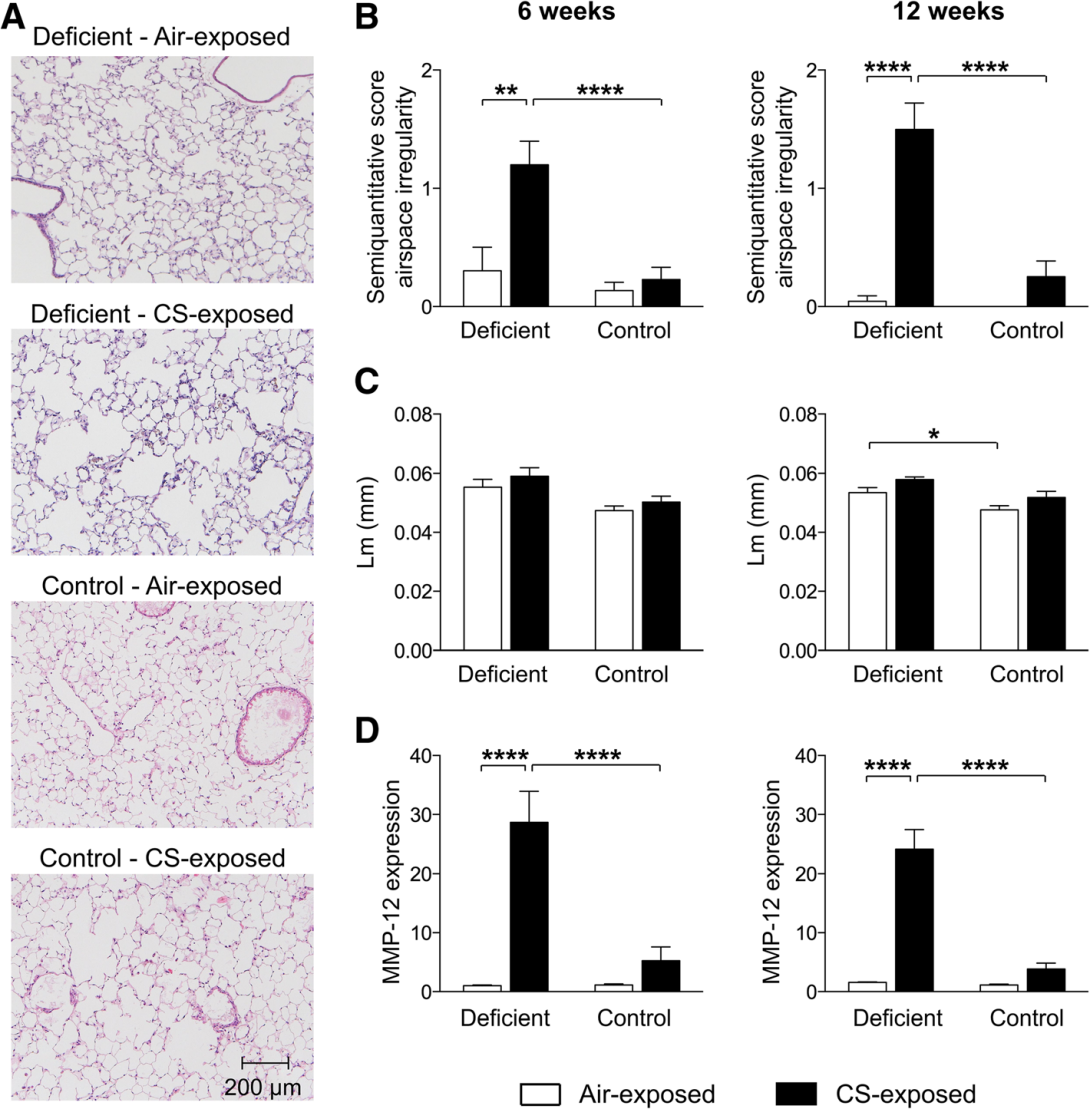
Effect of vitamin D deficiency on lung structure in air- and CS-exposed mice after 6 and 12 weeks. At the age of 8 weeks, C57Bl/6J vitamin D deficient and control mice were exposed to CS or ambient air for a period of 6 or 12 weeks. **a** Representative figures of H&E-stained lung sections of paraffinized lungs of each experimental group (100X magnification) after 6 weeks of smoking. **b** The irregularity of airspaces on histological lung sections was given a semi-quantitative score ranging from 0 to 3 by a pathologist in a blinded manner (0 = absent, 0.5 = minimal, 1 = mild, 2 = moderate and 3 = severe). **c** The mean linear intercept (Lm), as a measure of the interalveolar wall distance, was determined on histological lung sections (at 200X magnification). The Lm was measured in 15 randomly selected fields per lung slide and calculated as the total length of the grid lines x random fields divided by the sum of the alveolar intercepts. **d** Relative expression levels of MMP-12 in lung homogenates, analyzed with RT-PCR. Data were normalized using RPL-27 as housekeeping gene and analyzed with the comparative cycle threshold (Ct) method. *n* = 10–12 per group per timepoint; mean ± SEM; *p < 0.05, **p < 0.01, ****p < 0.0001

Figure 2a shows representative pictures of H&E-stained sections of paraffinized left lungs of each experimental group after 6 weeks of CS exposure. Emphysema was quantified on histological lung slices by measuring the mean linear intercept (Lm) as well as by scoring the irregularity of airspace sizes. Based on the semi-quantitative scores for the irregularity of airspace sizes, mild emphysema was found to be present in vitamin D deficient mice after already 6 and 12 weeks of smoking, but not in control mice (Fig. 2a-b). This was however not reflected by significant differences in Lm (Fig. 2c). Moreover, the increase in mRNA expression levels of macrophage elastase (MMP-12) due to CS exposure was more pronounced in vitamin D deficient mice compared to control mice (Fig. 2d). However, no differences in the mRNA expression of MMP-8, MMP-9 or TIMP-1 were observed (data not shown).

### Vitamin D deficiency exacerbates airway inflammation upon CS exposure

**Fig. 3:**
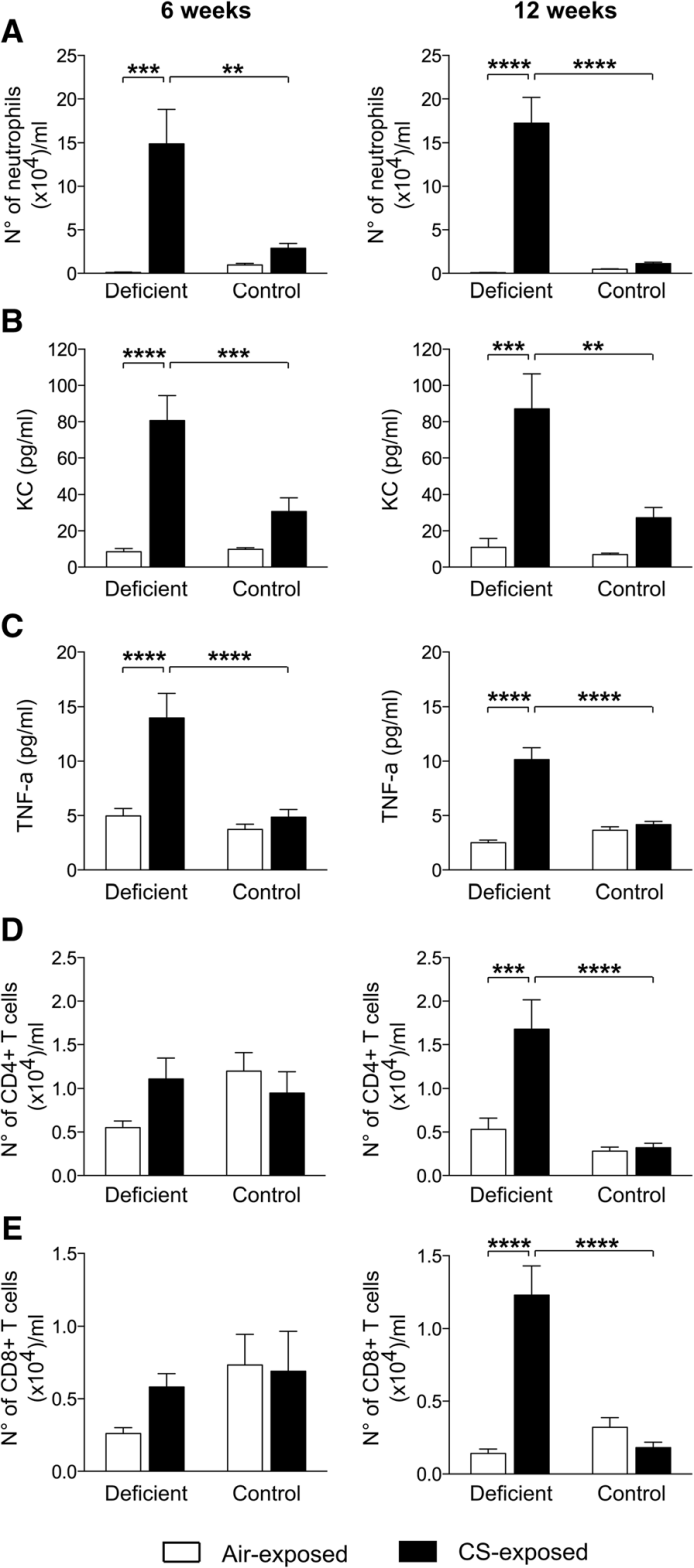
Effect of vitamin D deficiency on airway inflammation in air- and CS-exposed mice after 6 and 12 weeks. At the age of 8 weeks, C57Bl/6J vitamin D deficient and control mice were exposed to CS or ambient air for a period of 6 or 12 weeks. The number of **a** neutrophils (CD11c^−^CD11b^+^Ly6G^+^), **d** CD4^+^ T cells and **e** CD8^+^ T cells was determined in the BAL fluid using flow cytometry. Data are expressed as number of cells (×10^4^) per milliliter of recovered BAL fluid. Levels of **b** KC and **c** TNF-α were measured in the supernatant of the BAL fluid. *n* = 10–12 per group per time point; mean ± SEM; **p < 0.01, ***p < 0.001, ****p < 0.0001

We next assessed the cumulative effect of vitamin D deficiency on the size and composition of the airway inflammatory response to CS. Analysis of the bronchoalveolar cell infiltrate revealed no significant differences in the total cell number or in the number of CD11c^+^ autofluorescent^high^ alveolar macrophages after 6 and 12 weeks of smoking, independently of vitamin D status (data not shown). In control mice, a slight, non-significant increase in neutrophils was observed after 6 and 12 weeks of CS exposure (Fig. 3a). CS exposure dramatically exacerbated airway neutrophilia in vitamin D deficient mice compared to control mice to a similar extent at both subacute and chronic disease states. Moreover, the increase in levels of the neutrophil chemoattractant KC (mouse homolog of IL-8) as well as TNF-α in the BAL fluid following 6 and 12 week of CS exposure was more pronounced in vitamin D deficient mice compared to control mice (Fig. 3b-c). Protein levels of IL-6, IL-10 and IL-12 remained unchanged between the experimental groups (data not shown). Airway infiltration with T lymphocytes (CD4^+^ and CD8^+^ T cells) was only observed in vitamin D deficient mice after 12 weeks of smoking (Fig. 3d-e).

### Increased inflammation in the lung parenchyma of vitamin D deficient mice following CS exposure

**Fig. 4:**
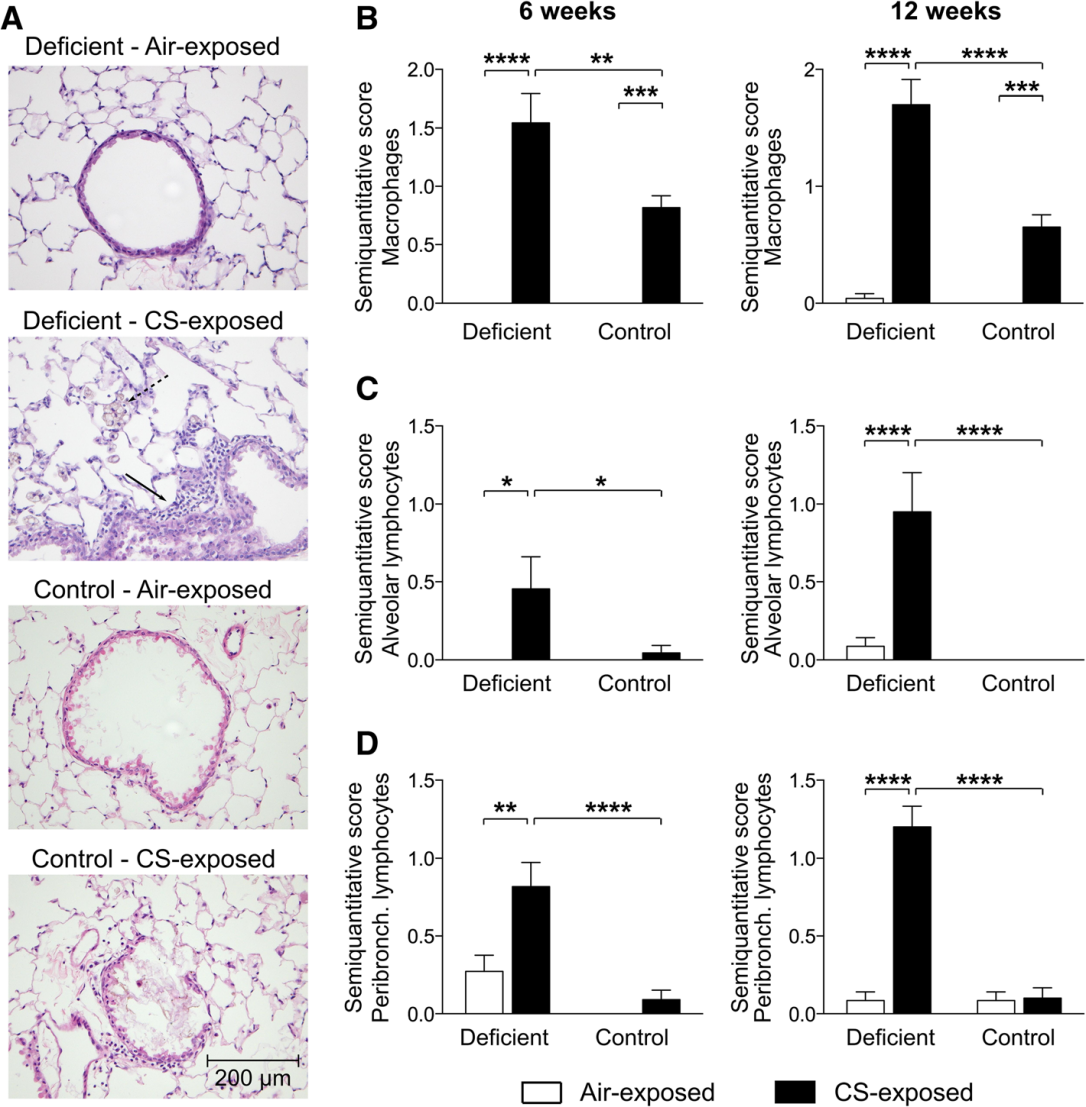
Effect of vitamin D deficiency on parenchymal inflammation in air- and CS-exposed mice after 6 and 12 weeks. At the age of 8 weeks, C57Bl/6J vitamin D deficient and control mice were exposed to CS or ambient air for a period of 6 or 12 weeks. **a** Representative figures of H&E-stained lung sections of each experimental group (200X magnification) after 6 weeks of smoking. Full arrows show the presence of peribronchial lymphocytic inflammation, while dashed arrows show the presence of pigmented macrophages in CS-exposed vitamin D deficient mice. **b** The diffuse presence of macrophages and the presence of **c** alveolar and **d** peribronchial lymphocytic inflammation on histological lung sections was given a semiquantitative score ranging from 0 to 3 by a pathologist in a blinded manner (0 = absent, 0.5 = minimal, 1 = mild, 2 = moderate and 3 = severe). *n* = 10–12 per group per time point; mean ± SEM; *p < 0.05, **p < 0.01, ***p < 0.001, ****p < 0.0001

**Fig. 5:**
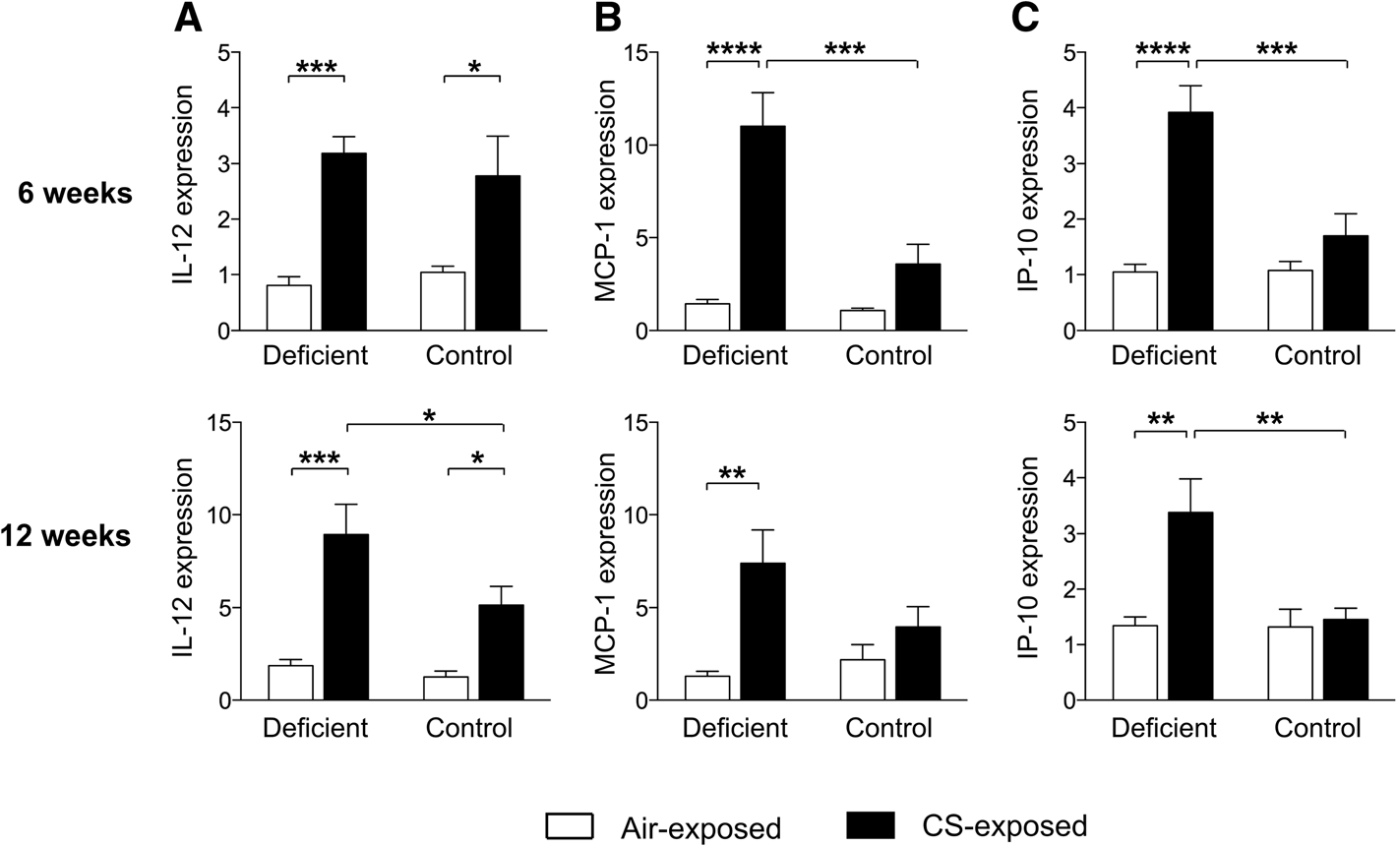
Effect of vitamin D deficiency on expression levels of proinflammatory cytokines and chemokines in lung tissue of air- and CS-exposed mice after 6 and 12 weeks. At the age of 8 weeks, C57Bl/6J vitamin D deficient and control mice were exposed to CS or ambient air for a period of 6 or 12 weeks. Relative expression levels of **a** IL-12, **b** MCP-1 and **c** IP-10 in lung homogenates were analyzed with RT-PCR. Data were normalized using RPL-27 as housekeeping gene and analyzed with the comparative cycle threshold (Ct) method. *n* = 10–12 per group per time point; mean ± SEM; *p < 0.05, **p < 0.01, ***p < 0.001, ****p < 0.0001

In addition to the extent of airway inflammation, parenchymal inflammation was evaluated on H&E-stained sections of paraffinized left lungs. Figure 4a shows representative figures of each experimental group after 6 weeks of CS exposure. A semiquantitative score was given based on the presence of macrophages, neutrophils or lymphocytes. In control mice, a mild increase in macrophages was observed in CS-exposed mice after 6 and 12 weeks of smoking (Fig. 4b). However, neutrophilic or lymphocytic infiltration, either peribronchial or alveolar, was not observed (Fig. 4c-d). Similar to control mice, neutrophilic inflammation was absent in the lung parenchyma of vitamin D deficient mice. However, the increase in the number of tissue macrophages due to CS exposure was much more pronounced in vitamin D deficient mice compared to control mice after 6 and 12 weeks (Fig. 4b). Low-grade peribronchial and alveolar lymphocytic inflammation could only be detected in vitamin D deficient mice after 6 and 12 weeks of smoking (Fig. 4a + c-d). As an additional parameter of inflammation within the interstitial lung tissue, relative mRNA levels of several inflammatory cytokines and chemokines were determined in right lung homogenates. Supporting a clear augmented inflammatory profile, the increase in expression levels of IL-12, MCP-1 and IP-10 following CS exposure was more pronounced in vitamin D deficient mice compared to control mice (Fig. 5a-c).

### Vitamin D deficiency only mildly affects CS–induced defects of alveolar macrophage antibacterial function

**Fig. 6:**
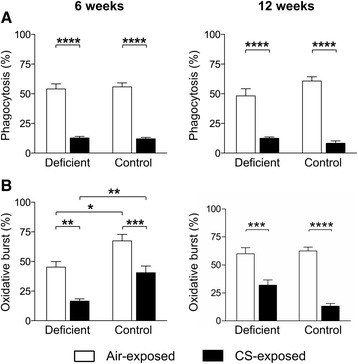
Effect of vitamin D deficiency on the antibacterial function of alveolar macrophages in air- and CS-exposed mice after 6 and 12 weeks. At the age of 8 weeks, C57Bl/6J vitamin D deficient and control mice were exposed to CS or ambient air for a period of 6 or 12 weeks. The *ex vivo* phagocytic and oxidative burst capacity of alveolar macrophages was assessed following interaction of BAL cells with *E. coli* bacteria. **a** Percentage of alveolar macrophages (CD11c^+^autofluorescent^high^) that have internalized *E. coli* bacteria. **b** Percentage of alveolar macrophages (CD11c^+^autofluorescent^high^) that have produced reactive oxygen radicals. *n* = 10–12 per group per time point; mean ± SEM; *p < 0.05, **p < 0.01, ***p < 0.001, ****p < 0.0001

To assess the effect of vitamin D deficiency on the antibacterial functionality of alveolar macrophages in the context of CS exposure, the *ex vivo* phagocytic and oxidative burst capacity of alveolar macrophages was assessed following interaction of BAL cells with *E. coli* bacteria. CS exposure significantly hampered the phagocytic capacity of alveolar macrophages to the same extent in both vitamin D deficient and control mice after 6 and 12 weeks of smoking (Fig. 6a). No additional effect of vitamin D deficiency was observed on the phagocytic capacity of alveolar macrophages. The production of reactive oxygen species was also decreased upon CS exposure in vitamin D deficient and control mice after 6 weeks (Fig. 6b). However, vitamin D deficiency triggered a slight further impairment of the CS-induced defect in oxidative burst capacity only at the subacute stage of the disease.

## Discussion

Vitamin D deficiency is highly common in COPD patients [3–5] and increasing evidence suggests a role for vitamin D deficiency in COPD pathogenesis, which is characterized by exacerbated airway and parenchymal inflammation as well as defective antibacterial responses. Despite many *in vitro* cell studies highlighting important anti-inflammatory and antimicrobial effects of vitamin D, evidence from epidemiological studies remains contradictory. Therefore, our aim was to investigate whether vitamin D deficiency can directly influence characteristic features of COPD pathogenesis in a mouse model of subacute and chronic CS exposure. We show that vitamin D deficiency accelerates lung function changes upon CS exposure consistent with an increased lung compliance and TLC, early signs of emphysema and enhanced expression of MMP-12. We furthermore demonstrate that vitamin D deficiency leads to exacerbated airway and parenchymal inflammation compared to control mice after both subacute and chronic CS exposure. CS exposure was associated with strong decreases in antibacterial functionality of alveolar macrophages. However, vitamin D deficiency did not affect bacterial phagocytosis in addition to CS, although minor additional defects were observed in the oxidative burst capacity after subacute CS exposure.

Whether vitamin D deficiency can directly influence lung function or emphysema progression in long-term smokers cannot be derived from observational studies as reversed causation, even with longitudinal design, cannot be excluded. Notwithstanding the shortcomings of mouse models with smoke-induced emphysema, they offer the advantage to explore causality by specific exposures. To our knowledge, only one study has investigated the effect of vitamin D deficiency on the development of emphysema in a mouse model of CS exposure [26]. Crane-Godreau *et al.* found more severe emphysema (as assessed by measurement of Lm) in vitamin D deficient mice compared to control mice after 16 weeks of smoking, without additional differences in lung function parameters (TLC and lung compliance). We extend on these data by showing that vitamin D deficiency accelerates lung disease progression upon CS exposure. In this context, hyperinflation of the lungs (as demonstrated by increases in TLC, compliance, IC, VC, FEV0.1 and FVC) was more pronounced in vitamin D deficient mice compared to control mice after already 6 weeks of smoking. However, after 12 weeks of smoking, hyperinflation of the lungs was also observed in control mice, without an additional effect of vitamin D deficiency. The increase in FEV0.1 and FVC with smoking is however contradictory to what is seen in human COPD but is explained by the compliant chest wall of mice, which allows an unlimited expansion of the lungs with positive pressure inflation and consequently higher volumes and flows during expiration [25]. We did not observe an effect of vitamin D deficiency on lung function parameters in air-exposed mice, in the absence of CS exposure after 6 or 12 weeks. This is in contrast to the findings of Zosky *et al.,* who found deficits in lung function in the offspring (2 weeks of age) of vitamin D deficient mice [27]. In our study, airspaces were more heterogeneously enlarged in vitamin D deficient mice after both subacute and chronic CS exposure compared to control mice. These early signs of emphysema may be (partially) explained by the increased expression of MMP-12 in vitamin D deficient mice following CS exposure. MMP-12 is a macrophage elastase which degrades the extracellular matrix and weakens the interstitial alveolar structure, and has shown to be increased in COPD patients [28, 29]. Furthermore, MMP-12 knockout mice were protected from CS-induced emphysema, demonstrating an important role for MMP-12 in the development of emphysema [30].

One of the hallmarks of COPD is the development of exaggerated chronic inflammation in the lungs in response to CS. This chronic inflammation is characterized by pulmonary infiltration of inflammatory cells, such as neutrophils, macrophages and lymphocytes, and elevated levels of (chemoattractant) cytokines, including TNF-α, IL-8, MCP-1 and IP-10 [31]. Interestingly, the active form of vitamin D, 1,25(OH)_2_D, has been shown to decrease the expression of these proinflammatory cytokines and chemokines in several cell types, including macrophages, neutrophils and airway epithelial cells [18–22, 32], potentially by interfering with the NF-κB and p38 MAP kinase inflammatory pathways. By downregulating the expression of inflammatory cytokines and chemokines, vitamin D could also indirectly influence the infiltration of inflammatory cells into the lungs. Using our mouse model, we show for the first time that CS exposure leads to exacerbated airway and parenchymal inflammation in vitamin D deficient mice compared to control mice, characterized by increased inflammatory cell infiltration and enhanced inflammatory mediators. The most prominent characteristic of the CS-induced airway inflammation was the neutrophilic infiltration, which was clearly more pronounced in vitamin D deficient mice and was accompanied by increased levels of KC and TNF-α. In a study of Li and colleagues, vitamin D deficiency also enhanced neutrophil infiltration into the lungs after challenge with *A. fumigatus* [33]. Furthermore, in a mouse model of allergic airway disease, it was shown that vitamin D deficiency enhances airway neutrophilic inflammation, which was subsequently suppressed after supplementation of vitamin D deficient mice with vitamin D [34]. In an animal model of acute lung injury, intratracheal or peroral administration of 1,25(OH)_2_D also inhibited the recruitment of neutrophils after LPS inhalation [35]. Knockout of the vitamin D receptor (VDR) in mice moreover resulted in increased inflammatory cell influx in the airways (neutrophils) and lung parenchyma (macrophages), which was accompanied by enhanced activity of NF-κB and elevated levels of MCP-1 and KC in the lungs [36]. In our study, CS exposure resulted in an increased number of macrophages in the lung parenchyma of vitamin D deficient mice compared to control mice. Furthermore, airway infiltration of CD4^+^ and CD8^+^ T cells and low-grade lymphocytic parenchymal inflammation was exclusively observed in CS-exposed vitamin D deficient mice. This was associated with increased expression of MCP-1 and IP-10 in lung tissue, which may explain the elevated infiltration of respectively mononuclear cells and lymphocytes in the lung parenchyma.

Defective antibacterial function of macrophages might contribute to the persistence of respiratory infections and consequently exacerbations in COPD patients [37, 38]. Several antibacterial functions have been attributed to 1,25(OH)_2_D, including stimulation of phagocytosis, oxidative burst, chemotaxis and the production of antimicrobial peptides *in vitro* [16, 17, 39, 40]. In our *in vivo* study we evaluated for the first time whether vitamin D deficiency can directly influence the antibacterial function of alveolar macrophages in CS-exposed lungs. Our data confirm that CS exposure leads to significant decreases in the *ex vivo* phagocytic as well as oxidative burst functions of alveolar macrophages. However, vitamin D deficiency did not further affect the phagocytic function of alveolar macrophages. Our data confirm the findings of Giuletti *et al.* in autoimmune diabetic NOD mice showing that vitamin D deficiency did not alter the *ex vivo* chemotactic or phagocytic capacity of peritoneal macrophages [41]. Indirectly, it may suggest that smoking, rather than a deficient vitamin D status, increases the risk for infection *in vivo,* which may explain why different human studies in COPD found no associations between 25OHD status and risk for exacerbations [12–14]. We do acknowledge that we solely assessed the *ex vivo* antibacterial functionality of alveolar macrophages and that *in vivo* bacterial challenge would offer more appropriate insights.

It should be noted that serum 25OHD levels in our mouse model are not unambiguously comparable to the human situation. In humans, serum 25OHD levels between 30 and 50 ng/ml are generally considered sufficient, although 25OHD levels necessary for extra-calcemic functions of vitamin D, including immunomodulation, are still debatable. However, in our study, C57Bl/6 mice on the control diet have serum 25OHD concentrations of approximately 75–90 ng/ml, which are already considerably higher than sufficient levels in humans. These relatively high 25OHD levels in control mice are comparable to other mouse studies, where no systemic side-effects of these 25OHD concentrations were observed [41, 42]. In humans, vitamin D deficiency is defined as serum 25OHD levels below 20 ng/ml, whereas levels below 10 ng/ml are considered severely deficient, which is about 4-fold lower than sufficient levels. In our mouse model, serum 25OHD levels in vitamin D deficient mice (17–20 ng/ml) were also approximately 4-fold lower compared to control mice (75–90 ng/ml). Therefore, the 25OHD levels reached in our vitamin D deficient mice may reflect a severely deficient status in mice, although these levels are higher than what other authors have used in models for vitamin D deficiency [27, 34, 41].

## Conclusions

In conclusion, we have shown that vitamin D deficiency accelerates and aggravates the development of COPD-like characteristics (lung function changes, emphysema and pulmonary inflammation) in the lungs following CS exposure, suggesting an important role for the vitamin D pathway in COPD pathogenesis and progression. Our data strengthen the epidemiological associations and mechanistic *in vitro* studies that link vitamin D deficiency to characteristic features of COPD. Two randomized placebo-controlled intervention trials have already shown the therapeutic benefit of vitamin D supplementation on exacerbation risk in COPD patients with vitamin D deficiency at baseline [43, 44]. As vitamin D deficiency is highly prevalent, large preventive trials assessing the effect of vitamin D supplementation on COPD onset will provide even more insights into the role of vitamin D deficiency in the development of COPD.

## Additional file

Additional file 1:
**The effect of vitamin D deficiency on additional lung function parameters in air- and CS-exposed mice after 6 and 12 weeks.** (PDF 96 kb)

## Abbreviations

1,25(OH)_2_D: 1,25-dihydroxyvitamin D
25OHD: 25-hydroxyvitamin D
BAL: Bronchoalveolar lavage
Cchord: Compliance
COPD: Chronic obstructive pulmonary disease
CS: Cigarette smoke
FEV0.1: Forced expiratory volume in 100 milliseconds
FEV1: Forced expiratory volume in 1 s
FVC: Forced vital capacity
IC: Inspiratory capacity
Lm: Mean linear intercept
MMP: Matrix metalloproteinase
TLC: Total lung capacity
VC: Vital capacity
