# Supplementary material for: Vitamin D deficiency exacerbates COPD-like characteristics in the lungs of cigarette smoke-exposed mice
Source: Respir Res. 2015 Sep 16;16(1):110. doi: 10.1186/s12931-015-0271-x (PMC4574263; doi:10.1186/s12931-015-0271-x)
Supplement: Additional file 1: — The effect of vitamin D deficiency on additional lung function parameters in air- and CS-exposed mice after 6 and 12 weeks. (PDF 96 kb) [file 12931_2015_271_MOESM1_ESM.pdf]

Additional file 1

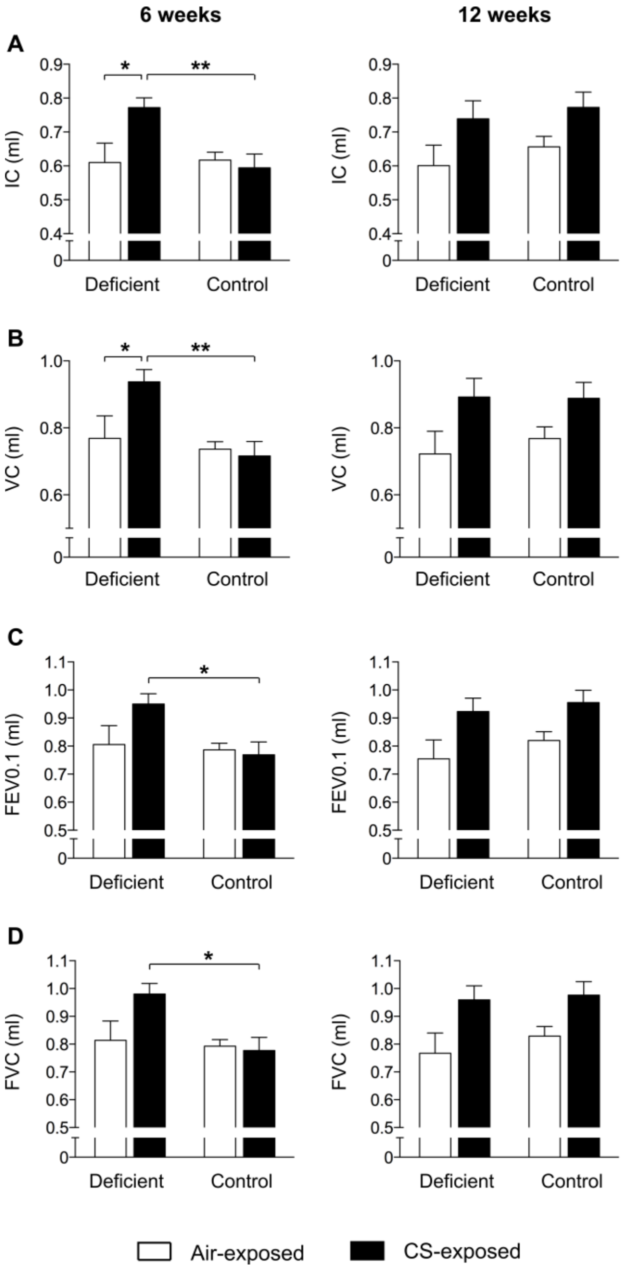

Effect of vitamin D deficiency on additional lung function parameters in air- and CS-exposed mice after 6 and 12 weeks. At the age of 8 weeks, C57Bl/6J vitamin D deficient or control mice were exposed to CS or ambient air for a period of 6 or 12 weeks. Lung function was measured with whole-body plethysmography after 6 and 12 weeks of smoking. **(A)** Inspiratory capacity (IC), **(B)** Vital capacity (VC), **(C)** Forced expiratory volume in 100 milliseconds (FEV0.1) and **(D)** Forced vital capacity (FVC). n=10-12 per group per time point; mean±SEM; *p<0.05, **p<0.01.
